# Transport Properties of Electro-Sprayed Polytetrafluoroethylene Fibrous Layer Filled with Aerogels/Phase Change Materials

**DOI:** 10.3390/nano10102042

**Published:** 2020-10-16

**Authors:** Xiaoman Xiong, Mohanapriya Venkataraman, Tao Yang, Klara Kucerova, Jiří Militký, Kai Yang, Guocheng Zhu, Juming Yao

**Affiliations:** 1 Department of Material Engineering, Faculty of Textile Engineering, Technical University of Liberec, 46117 Liberec, Czech Republic; mohanapriya.venkataraman@tul.cz (M.V.); Jiri.Militky@tul.cz (J.M.); kai.yang@tul.cz (K.Y.); 2 Institute for Nanomaterials, Advanced Technologies and Innovation, Technical University of Liberec, 46117 Liberec, Czech Republic; tao.yang@tul.cz (T.Y.); klara.kucerova2@tul.cz (K.K.); 3 School of Material Science and Engineering, Zhejiang Sci-Tech University, Xiasha Higher Education Zone, Hangzhou 310018, China; guocheng-zhu@hotmail.com (G.Z.); yaoj@zstu.edu.cn (J.Y.)

**Keywords:** polytetrafluoroethylene, silica aerogel, phase change material, electrospray, thermal properties, water vapor permeability

## Abstract

This work is the first attempt to prepare microporous polytetrafluoroethylene (PTFE) fibrous layers embedded with aerogels/phase change materials. For preparation of this layer, the needle-less electrospray technology of water dispersion of individual components is used. Microstructure characteristics, including surface morphology and particle size distribution, and various properties of the prepared materials were investigated and explained. Transport performance of the fibrous layers embedded with aerogels/phase change materials, such as the transmission of heat, air, and water vapor was evaluated and discussed in details. It was found that the electro-sprayed materials composed by spherical particles with rough surface had compact disordered stacking structure. Aerogels and phase change materials (PCMs) play different roles in determining structural parameters and transport properties of the materials. Those parameters and properties could be flexibly adjusted by optimizing the spinning parameters, changing the content or proportion of the fillers to meet specific requirements.

## 1. Introduction

Polytetrafluoroethylene (PTFE) has many unique characteristics such as outstanding chemical stability, non-stick property, corrosion resistance, low thermal conductivity, good thermal stability under high temperature, strong hydrophobicity and high fracture toughness, as a result of the strong C–C bonds in the carbon backbone and side-F groups responsible for the uniform helical sheath formed by the electron cloud of the fluorine atoms [1]. These properties make PTFE attractive for various engineering applications. However, due to its low solubility to most of the solvents and high melt viscosity, it is impossible to fabricate PTFE porous materials by using of some conventional methods, such as phase inversion process and melting-stretching technique.

Electrospray is a promising and attractive approach to develop PTFE porous materials with the advantage of easy management and good flexibility of obtaining a variety of materials to meet different requirements. Electrospray produces small monodisperse particles, in the form of a thin film of fine particles, from a colloidal suspension of solid nanoparticles or a solution of a material. An electrospray uses large electric fields to generate a spray of highly charged droplets, the emitter from which the spray originates is a small diameter capillary outlet connected to a high voltage source relative to a ground electrode positioned in front of the emitter [2]. As the electric field becomes stronger, fluid at the edge of the capillary outlet deforms into a Taylor cone jet. If the spinning fluid has sufficiently high surface tension and lower viscosity the jet disintegrates downstream and subsequently breaks up into charged droplets, forming a spray. These droplets are sub-micrometer or micro-meter in diameter and they rapidly evaporate due to their large surface-to-volume ratio, forming an electro-sprayed particle layer deposited on a substrate. Electrospray enables the use of various fillers for performance modification, offering more possibility for the development of PTFE-based porous materials. Properties of these composites strongly depend on the intrinsic properties and interface performance of the polymer and the fillers used [3], the content, size, and mass fraction of fillers [4,5].

Silica aerogel and phase change material (PCM) are two types of particularly interesting materials widely used as fillers in fibrous materials or composites to improve thermal properties. Silica aerogel, as a gel composed of a porous solid where the dispersed phase is air, exhibits lower thermal conductivity than air under the same condition [6] and thus has the potential for low-weight composites presenting extraordinary thermal and multifunctional properties [7]. The lightweight silica aerogel particles have been coated on cotton woven fabric [8] and wool-Aramid blended fabric [9], incorporated with polyester nonwovens by thermal bonding [10], encapsulated in a multi-layered composites [11], embedded in nanofibrous structure by electrospinning [12], successfully achieving significantly lower thermal conductivity of the overall structures. PCMs, functioning by absorbing external incoming heat flux due to phase change and acting as thermal buffering of sudden heat changes, were used for several years mainly for thermal regulating purposes. Organic PCMs with wide range phase transition from 18 till 65 °C are suitable for thermal applications as they show consistency in repeated phase changes [13] and offer a small temporary heating or cooling effect. The temporary enhancement of thermal protection by using PCMs in firefighter’s garment was extensively proved by many researchers [14,15,16]. The simultaneous use of aerogels and PCMs coated on a multilayer fabric structure in firefighter’s protective garment provided superior thermal protection and comfort while the final performance of the proposed combination depends on the amount of PCM and its melting enthalpy [13].

A considerable amount of literature has been published on PTFE porous materials, such as the ultrafine fibrous PTFE porous material [17] and PTFE hollow fiber membrane [18] fabricated by electrospinning, focusing on investigation of surface properties. Publications that concentrate on PTFE/aerogel composites more frequently adopt dry mixing of PTFE particles with aerogels [19], wet mixing using aqueous PTFE dispersion [5] or PTFE powder [7,20] for engineering applications. Applying electrospray technique to fabricate PTFE porous materials has been carried out to investigate superhydrophobic behavior of the materials [21,22]. Previous research on electro-sprayed PTFE layers filled with carbon microparticles emphasized the surface properties including roughness, electrical property, and hydrophobicity [23]. So far, however, there has been no attempts to fabricate aerogel or PCM embedded PTFE microporous materials by using electro-spray technique. Transport performance of electro-sprayed PTFE porous materials, such as the transmission of heat, air, and water vapor, still remains unclear. The objective of this study is to fabricate PTFE porous materials embedded with aerogels/PCMs via needle-less electrospray technology and investigate the microstructure characteristics as well as various transport properties. This work could be an important contribution to the field of development and performance modification of PTFE porous materials. It could help further understand the structural characteristic of electro-sprayed PTFE materials and the effect of aerogels, PCMs, as well as the coupled aerogels and PCMs, on transport properties of the overall structure. The prepared materials have the potential to be used for various applications including protective clothing and barrier sheets, by optimizing the spinning liquid composition and also the spinning parameters.

## 2. Methodology

### 2.1. Materials

The basic ingredient for electrospray is a commercially available aqueous dispersion of the polymer named Teflon^TM^ PTFE DISP 30 (Chemours, Wilmington, NC, USA). This product is a fluoropolymer suspension containing 60% by weight of polytetrafluoroethylene with typical particle size 220 nm, suspended in distilled water, together with a 6% by weight surfactant. Density of the dispersion is 1510 kg/m^3^, the viscosity at 25 °C is 25 MPa s. High-purity (99.999%) silica aerogels in the form of powders and granules were both purchased from Cabot Corporation (Boston, MA, USA) to composite with the PTFE via electro-spraying. Specifications of the aerogel powders are listed in Table 1. These silica aerogels are selected because they are commercially available and widely used for insulative coating applications with various functional advantages such as ultra-low thermal conductivity, fully hydrophobicity, safe touch performance and smooth finish. Since the particle size of aerogels may influence the composition and final performance of the electro-sprayed materials, aerogel granules with majority particle size ranging from 0.1 to 0.7 mm were used for comparison. Besides, PCMs in the form of microcapsules synthesized by Zhejiang Sci-tech University (Hangzhou, China) were selected as filler as well. The PCMs were prepared via mixing the oil-phase solution consisted by n-octadecane, methyl methacrylate, butyl acrylate, 2,2′-azobisisobutyronitrile and 2-hydroxyethyl methacrylate with sodium a-olefinsulfonate aqueous solution, shearing and emulsifying the two-phase system, heating and stirring, followed by polymerization [24]. The microcapsules core PCM material is n-octadecane with a general formula C_n_H_2n+2_ and shell material is polymethyl methacrylate known as (C_5_O_2_H_8_)_n_ [24]. Particle size of the PCMs microcapsules ranged from 91.28 to 1484 nm, with a mean value 826 nm. Density of the PCM microcapsules was about 780 kg/m^3^. These PCM microcapsules were spherical with a smooth surface, offering larger surface tension and better dispersity for further processing and application. The melting temperature range of microcapsules PCM core was 24.62–28.71 °C [24]. All the mentioned materials were used as received.

### 2.2. Fabrication of Electro-Sprayed PTFE Embedded with Aerogel/PCM

**Figure 1:**
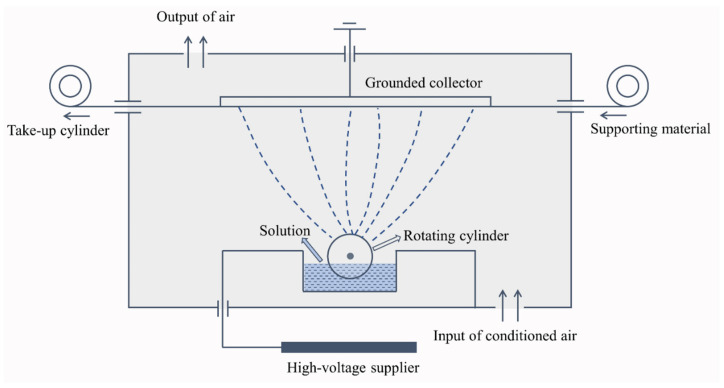
Schematic diagram of needle-less electrospray.

A certain quantity of fillers (aerogels or PCMs) was weighted and mixed with PTFE dispersion to prepare spinning liquid with a specific filler concentration. The liquid was stirred 2 h at room temperature to achieve homogeneous dispersions. Any visible deposition was avoided. The liquid was subsequently transferred to the solution tank in Nanospider device NS 1WS500U (Elmarco Inc., Morrisville, NC, USA) for electro-spraying. The electrode distance was 125 mm, the electrode rotation speed was set at 8 rpm, the applied voltage was −10/45 kV, and the airflow supply was 90/100 m^3^/h. A polypropylene (PP) spun-bond nonwoven fabric was used as supporting material, the delivery speed was 15 mm/min. The temperature and relative humidity were respectively kept at (22 ± 1) °C and (40 ± 2)% during the entire electrospray process. These spinning parameters were determined based on previous trials, which could ensure continuous spinning to prepare even materials. Schematic diagram of the needle-less electrospinning system of Nanospider used for electrospray is illustrated in Figure 1. The electrostatic force produced between the high voltage supplier and the grounded collector draws the liquid from the surface of electro rotating cylinder, the charged jet then breaks down into small droplets that solidify during the course and are collected on the surface of support material due to the sufficiently low liquid viscosity [25]. Since the liquid jets are not continuous, the formed film consists of small particles. The electro rotating cylinder keeps the ongoing electrospray process to produce the particles and the even layer of particles is obtained with coordination of the movement of support material.

After electro-spraying, the PP nonwoven fabric sheet with electro-sprayed particle surface was dried at 105 °C for 20 min. Besides applying a single type of filler, aerogel particles and PCMs were simultaneously used to prepare electro-sprayed PTFE filled with both aerogels and PCMs, following the same procedure as described. A sample without any fillers was prepared as well. The spinning liquids composition for individual samples is shown in Table 2.

## 3. Characterization

### 3.1. SEM-EDX

Scanning electron microscope (SEM) images were obtained after coating the samples with a gold film, using a VEGA (TESCAN Inc., Warrendale, PA, USA) operating at 30 kV, which provides detailed high-resolution images of the electro-sprayed particles by a focused electron beam across the surface and detecting secondary or backscattered electron signal. The SEM spectrometer was equipped with Energy Dispersive X-Ray (EDX) attachment with Oxford Instruments X-Max^N^ Silicon Drift Detector (SDD), enabling performing elemental chemical analysis of the electro-sprayed materials. The EDX measurements were acquired from the characteristic peaks of elements present in the materials via Aztec software (Oxford Instruments, Concord, MA, USA).

### 3.2. Particle Size Distribution

Based on the SEM images, size distribution of the filler particles was determined via image analysis with Image Tool software. This micro level image analysis is a convenient and effective approach to detect the fillers size and size distribution, dealing with morphological studies in various fields.

### 3.3. Thickness and Areal Density of the Materials

Thickness of the prepared materials was determined according to the standard ASTM D1777-15 using a fabric thickness tester. Weight per unit length (GSM) of the materials was measured following ASTM D3776-96 standard.

### 3.4. Air Permeability

Fx-3300 air permeability tester (TESTEST AG, Schwerzenbach, Switzerland) was used to measure air permeability of the electro-sprayed materials following ISO 9237 standard, Determination of the Permeability of Fabrics to Air. Air permeability is described as the ability of a material to transmit air when a certain air pressure drop is applied on both surfaces of the material. Pressure drop 200 Pa was used for the measurements. Each sample was measured 10 times and the values were averaged.

### 3.5. Thermal Properties from Alambeta

Thermal conductivity and thermal resistance of the electro-sprayed materials were evaluated by using Alambeta Instrument (SENSORA, Liberec, Czech Republic) according to EN 31092 Standard. The measuring head of the Alambeta moves down to contact with the specimen which is placed on the flat measuring plate, the copper block present in the measuring head is maintained at 32 °C by a thermometer connected to the regulator, the thermal drop between the two surfaces of the specimen is recorded with direct heat flow sensor [26]. According to the Fourier’s law of heat conduction, values of thermal conductivity and thermal resistance are automatically calculated and showed on the display screen.

### 3.6. Water Vapor Permeability

Permetest Instrument (SENSORA, Liberec, Czech Republic) was employed for the determination of relative water vapor permeability (%) and evaporation resistance *R_et_* (m^2^ Pa/W). The most important component of this device is the measuring head which is covered by semi-permeable foil to keep the sample dry. The heat flow value without a sample is evaluated, and then the fabric was inserted between the measuring head and the orifice in the bottom of the channel to test the heat loses of measuring head covered by a sample. As defined in ISO 11092, water vapor resistance *R_et_* is expressed as

(1) $$R_{et}=\frac{P_{m}-P_{a}}{q_{s}^{-1}-q_{0}^{-1}}$$

where *P_m_* is partial pressure [Pa] of the saturated water vapor of the ambient temperature, *P_a_* is water vapor partial pressure [Pa] of the room, *q_s_* is the heat loss [W/m^2^] of measuring head covered by a sample, *q*_0_ is the heat loss [W/m^2^] of measuring head without a sample.

## 4. Results and Discussion

### 4.1. SEM Analysis of the Electro-Sprayed Materials

**Figure 2:**
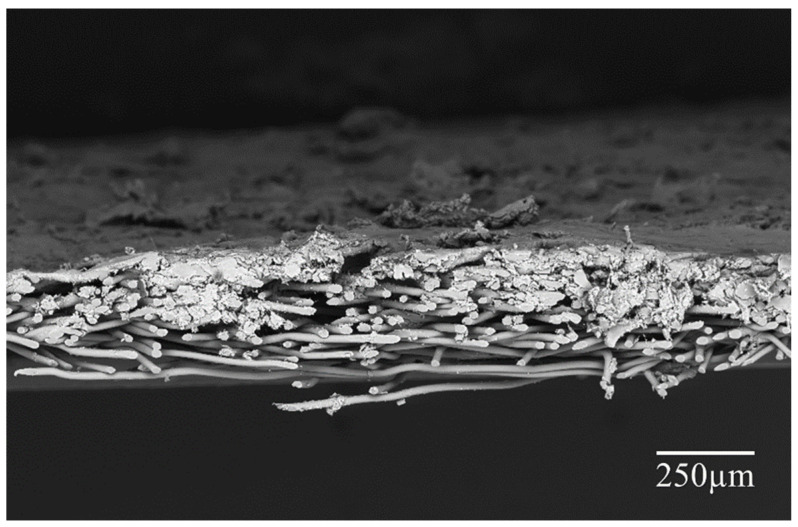
Typical cross section image of the electro-sprayed material.

**Figure 3:**
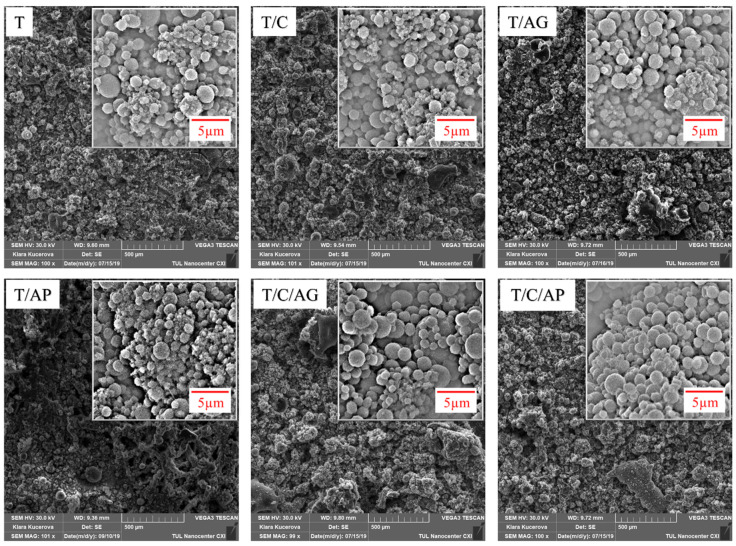
SEMs of the electro-sprayed materials with low magnification (100×) and high magnification (10,000×).

As seen in Figure 2, the electro-sprayed particles successfully deposited on the nonwoven substrate, forming a multi-layered cross section structure. The surface morphology of each material is illustrated in Figure 3. According to the images, the Nanospider technique for this dispersion produced droplets which form spherical solid particles uniformly distributed on the substrate surface. Apparently, the electro-sprayed particles were mostly in spherical shape with rough surface. These particles with different dimensions, mainly in microscale, gathered and partly overlapped with each other, forming a microporous overall structure. The visible single particle is actually a gathering of several small particles, where smaller particles attached on larger ones, exhibiting a compact disordered stacking structure. Aerogels, PCMs, and their combination had insignificant effect on the shape and arrangement of these electro-sprayed particles. However, as the filler used in each material varies, these materials showed slightly different overall surface morphology. Sample T/C/AG and T/C/AP demonstrated more compact structures with smaller particle size variations.

### 4.2. EDX Analysis of the Electro-Sprayed Materials

**Figure 4:**
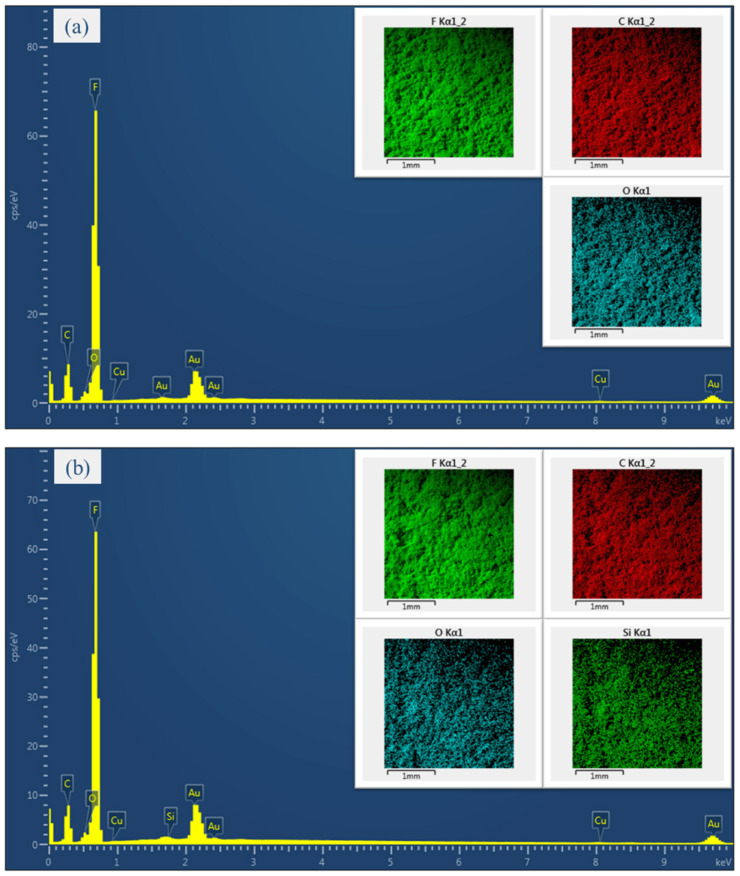

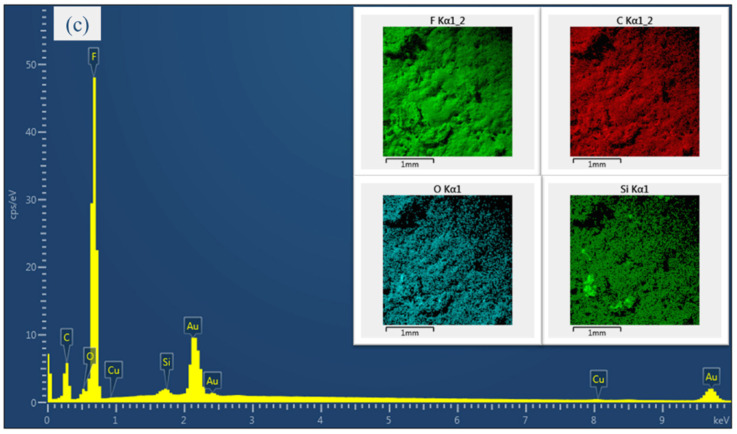
Typical X-ray spectrum and element maps of the electro-sprayed materials (**a**) sample T/C; (**b**) sample T/AG; (**c**) sample T/C/AP.

The multiple elements in the electro-sprayed materials and element maps obtained from EDX measurement are illustrated in Figure 4. The Au element is attributed to the gold coating necessary for the measurement, while the F element is originated from PTFE and the Si element is the key characteristics of silica aerogels. Typical element features of the materials without any aerogels are shown as sample T/C. According to the characteristic peaks, the elements F, C and O can be considered to be major elements. The highest peaks observed from F indicates an overwhelmingly dominance of PTFE. The element maps reveal an even distribution of each composition over the entire measuring area. The small amount of silica element appeared on sample T/AG proves the presence of aerogels in the electro-sprayed layer. The uniformly distributed F, C, O and Si element also confirm the homogeneity of the composite. Sample T/C/AP is observed to have similar element characteristic with sample T/AG. However, the O and Si element are found to have higher concentration in this material, indicating more aerogels loaded on the surface. The Cu element is probably resulted from the surfactant presented in PTFE dispersion.

### 4.3. Particle Size Distribution of Electro-Sprayed Materials

**Figure 5:**
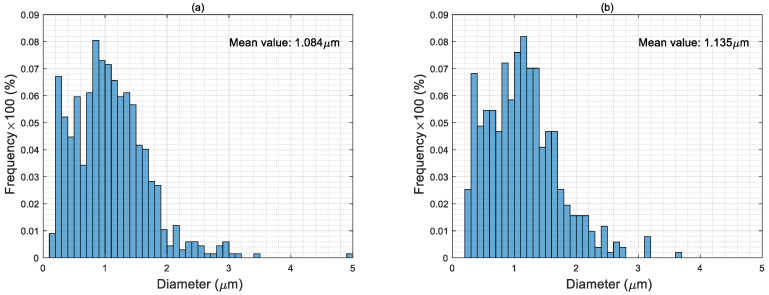

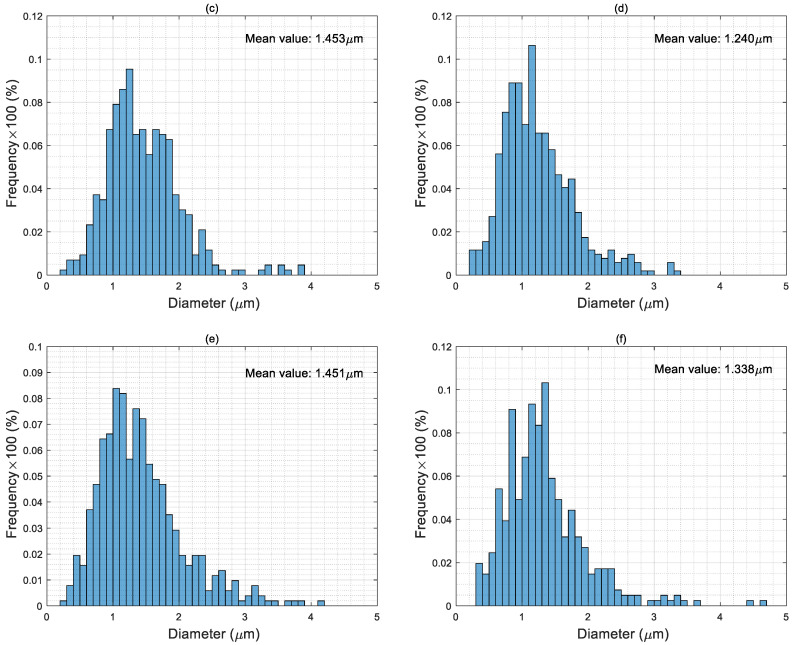
Particle size distributions of the electro-sprayed materials (**a**) sample T; (**b**) sample T/C; (**c**) sample T/AG; (**d**) sample T/AP; (**e**) sample T/C/AG; and (**f**) sample T/C/AP.

As seen in Figure 5, the particle diameters of pure PTFE microporous materials are mostly in the range of 0.4–1.8 μm, with a mean value 1.084 μm, which is the smallest among all the samples. It should be noticed that the particle size of PTFE solid present in the suspension is only around 220 nm, but the mean value of the electro-sprayed particles is several times larger. This further indicated that the observed PTFE particles in the microporous materials are coagulations of droplets capturing several single particles. In electrospray process, the PTFE particles travel with the liquid and get encapsulated inside the droplets after the jet breakup. The emitted droplets undergo solvent evaporation, these droplets disrupt to generate smaller droplets containing fewer or even one single particle in one drop if the electrostatic force is sufficiently strong to overcome surface tension [27,28]. Therefore, the surface charge density of the droplet plays an important role in size distribution of the electro-sprayed particles. The slightly larger average particle dimension in sample T/C is probably attributed to the change of conductivity of the liquid, which influences surface charge density of the droplets as well as the content and distribution of PCMs in the material. Applying aerogels in the spinning liquid could significantly increase the proportion of particles over 2 μm and remarkably decreases small particles under 0.6 μm since the fully hydrophobic surface of aerogels affects the charge density of the droplets and the distribution of different particles. Considering the simultaneous use of aerogels and PCMs, the particle size distributions of these materials are quite close to the samples with only aerogels.

### 4.4. Effect of Aerogels and PCMs on Thickness and Areal Density of the Materials

**Figure 6:**
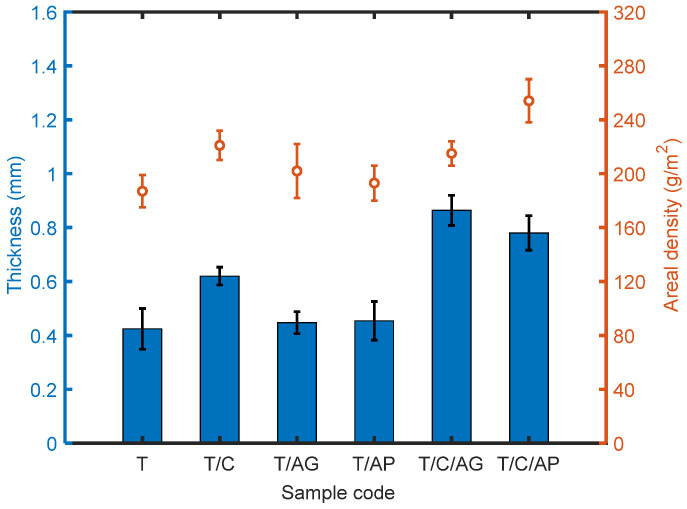
Thickness and areal density of the electro-sprayed materials.

Thickness and weight of the multi-layered materials are presented in Figure 6. The specimen containing PTFE microparticles has a thickness of 0.424 mm, while the materials consisting aerogels (sample T/AG and T/AP) were only slightly thicker despite the considerably bigger size of aerogels in comparison with PTFE particles. This is probably due to the change of viscosity and electrical conductivity of the solution after adding aerogels, which causes less supply of the particles during electrospray. The significant increase of thickness for those materials containing PCMs is resulted from the hydrophilic surface of PCMs, which improves the ejection of the liquid and therefore create more microparticles. In the case of material weight, PCMs applied in the spinning liquid cause considerable weight increase for the final materials. Besides, both the aerogel granules and aerogel powders decrease the material weight as expected. These materials can be used in applications where less weight burden is necessary.

### 4.5. Effect of Aerogels and PCMs on Air Permeability

**Figure 7:**
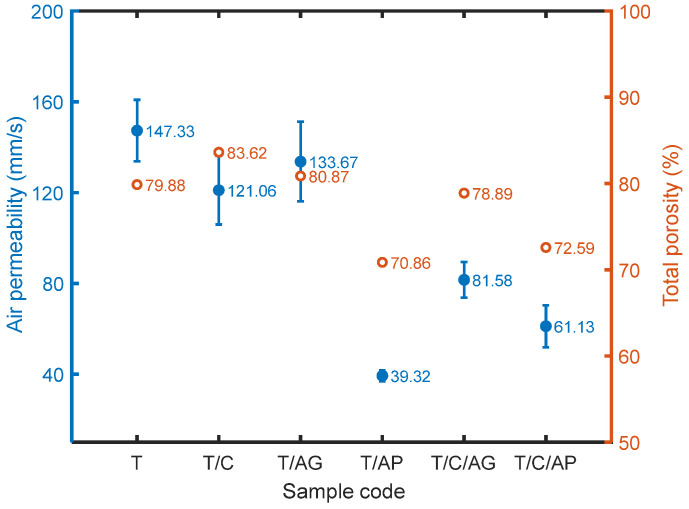
Air permeability and total porosity of the electro-sprayed materials.

Air permeability directly depends on pore size and porosity. All the materials are air permeable as seen in Figure 7, which is mainly attributed to the microporous characteristic of the materials. The highest air permeability value of sample *T* and relatively lower values of other samples revealed that aerogels and PCMs affect air permeable properties of the electro-sprayed materials. Aerogels could be approximately considered to be air-proof material, therefore, materials containing aerogels showed lower air permeability in comparison with sample *T*. In particular, aerogel powder has smaller particle size with less pores, which is able to be well incorporated with PTFE microparticles and thus dramatically increases the resistance to airflow. Since the PCM particle inherently has a solid body without any pores, its present in the overall structure leads to lower air permeability as well.

The total volume porosity *P*_0_ [−] of layers assembly is defined as

(2) $$P_{0}=1-\frac{w_{T}}{h\cdot \rho_{F}}$$

where $w_{T}$ [kg/m^2^] is planar mass (usually in *gsm*/1000, where *gsm* is grams of layer per surface area in square meter), h [m] is layer thickness and $\rho_{F}\;$ [kg/m^3^] is layer solid phase density. This density is calculated from expression

(3) $$\rho_{F}={\left(\frac{w_{A}}{\rho_{A}}+\frac{w_{PCM}}{\rho_{PCM}}+\frac{w_{PTFE}}{\rho_{PTFE}}+\frac{w_{PP}}{\rho_{PP}}\right)}^{-1}$$

where $w_{A}$, $w_{PCM}$, $\;w_{PTFE}$, $w_{PP}$ are mass fractions of aerogel, PCM, PTFE, PP and $\rho_{A}$ = 120~150 kg/m^3^, $\rho_{PCM}$ = 780 kg/m^3^, $\rho_{PTFE}$ = 2200 kg/m^3^, $\;\rho_{PP}$ = 920 kg/m^3^ are corresponding densities. Calculated values of *P*_0_ are shown in Figure 6. As the total porosity decreases, air permeability of the multilayered materials tends to decrease as well.

### 4.6. Effect of Aerogels and PCMs on Thermal Comfort Properties

**Figure 8:**
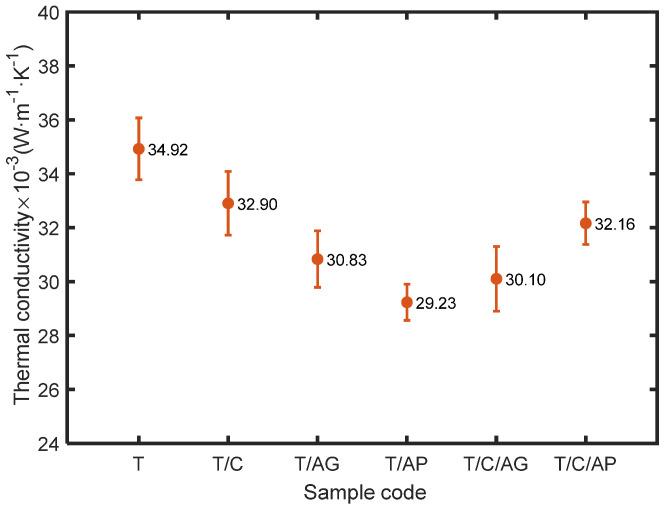
Thermal conductivity of the electro-sprayed materials.

Thermal conductivity, measuring the rate at which heat is transferred through unit area of the material across unit thickness under a specified temperature gradient, is an inherent property of a material. As shown in Figure 8, the PCMs present in the electro-sprayed PTFE materials contribute to a lower thermal conductivity probably by means of lower thermal conductivity of PCM shell (polymethylmetacrylate thermal conductivity is 0.19 W m^−1^ K^−1^) in comparison with Teflon (0.25 W m^−1^ K^−1^). Very low thermal conductivity was achieved by applying aerogels as seen for sample T/AG and T/AP. In particular, sample T/AP has a thermal conductivity as low as 0.029 W m^−1^ K^−1^, behaving better than most of the existing porous insulation materials. Generally, conventional porous or fibrous insulation materials such as polyurethane foam, fiber assembly or fabrics made by various natural or man-made fibers rely on the component of stagnant air in the structure to resist heat transfer through because stagnant air has several times lower thermal conductivity value (0.024 W m^−1^ K^−1^) than most of the solid and liquid matters. By adjusting the structure characteristics especially overall porosity, conventional porous materials could achieve a thermal conductivity comparable to 0.030 W m^−1^ K^−1^, which could be classified as good insulators since their thermal conductivity is less than 0.033 W m^−1^ K^−^^1^. However, these materials are several milli-meters or centi-meters thick, which are applicable for engineering use. The electro-sprayed materials have the advantage of low thermal conductivity as well as very small thickness, showing the potential to be used in protective clothing and barrier sheets where the material thickness is greatly restricted. It is also noted that aerogel powder is superior to aerogel granule for decreasing heat transfer rate in this work. This is probably benefited from the smaller particle size which enables the formation of more even material with higher content of aerogels, resulting in lower heat transfer rate. The simultaneous use of aerogels and PCMs leads to slightly lower thermal conductivity than sample T/C, but there is no significant advantage when compared to sample T/AP and T/AG.

**Figure 9:**
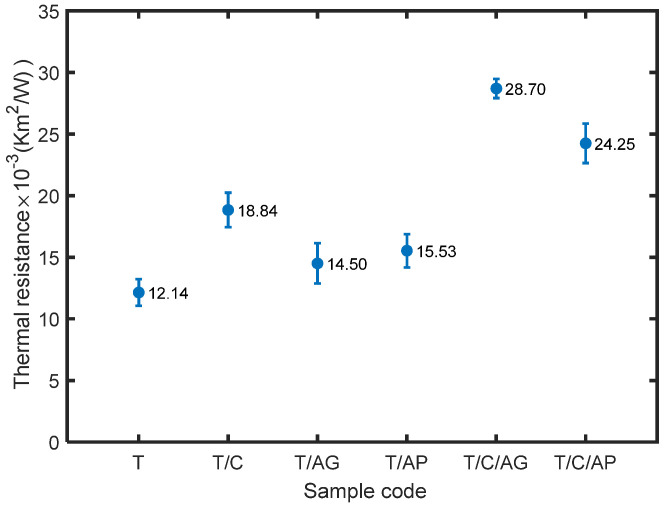
Thermal resistance of the electro-sprayed materials.

Thermal resistance is determined by thermal conductivity and thickness of the material, expressing the ability of a material to prevent heat flow through the thickness over unit surface area. Figure 9 shows thermal resistance of the prepared materials. Aerogels in the form of powder and granule were both able to achieve slightly thicker materials with higher thermal resistance when compared to sample T. Incorporation of PTFE with PCMs and aerogels (powder or granules) could create much thicker materials with significantly enhanced thermal insulation ability. However, since the electro-sprayed materials are very thin, they could be used as liner in garment or glove to provide better insulation behavior.

### 4.7. Effect of Aerogels and PCMs on Water Vapor Transmission

**Figure 10:**
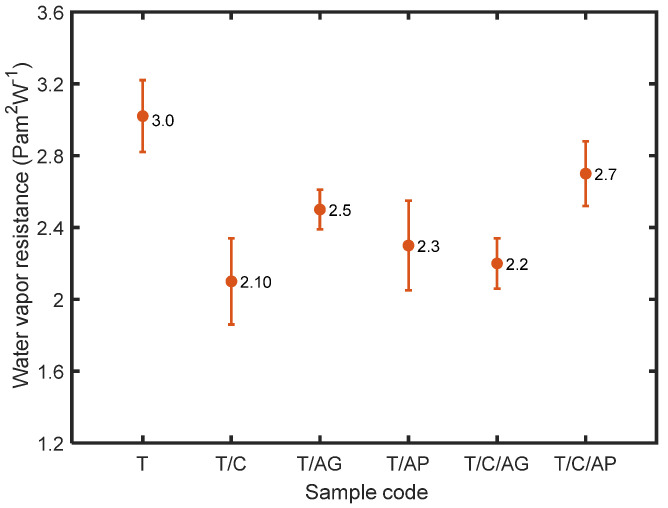
Water vapor resistance of the electro-sprayed materials.

As a measure of the passage of water vapor through the material, water vapor permeability depends on the water vapor resistance which indicates the amount of resistance against the transport of moisture through the structure. All the materials are extremely water vapor permeable with water vapor resistance less than 6 m^2^ Pa/W as seen in Figure 10. The electro-sprayed PTFE materials, consisting of numerous tiny interconnected pores, are hydrophobic to prevent liquid water from wetting the surface and entering the pores [29]. Since the sorption of water vapor does not occur to any appreciable extent, its water vapor transmission depends on the pathway of moisture vapor diffusion through the air spaces in the materials. Aerogels, present in the electro-sprayed materials, could alter the pore characteristic of the overall structure, thus improve the moisture transmission and decrease the water vapor resistance. Materials that incorporate hydrophilic particles (PCMs) behave quite differently than materials that depend only on transport through air spaces. The permeability coefficient for hydrophilic particles increases with material thickness, the affinity of the water molecules leads to significant water vapor sorption as well, resulting in significant increase in water vapor permeability [30]. For materials containing PCMs, the resistance to water vapor transmission is independent of the water vapor concentration in the material, but strongly influenced by the relative humidity on both sides of the sample. Therefore, smaller values of water vapor resistance are observed for these materials.

## 5. Conclusions

Microporous PTFE materials embedded with aerogels/PCMs were prepared via Nanospider technology to investigate various transport properties. It was found that the created materials composed by spherical solid particles had a compact disordered stacking structure. The fillers had insignificant effect on the particle shape and surface morphology of the overall structure, but affected size distribution of the electro-sprayed particles. Results also showed that aerogels could dramatically decrease heat transfer rate of the electro-sprayed materials and slightly increase water vapor transmission by means of influencing the pore characteristic of the overall structure while the PCMs contributed to thicker electro-sprayed materials with better thermal insulation ability and significantly improved water vapor permeability. Therefore, aerogels and PCMs fillers are beneficial for preparing electro-sprayed materials for protective clothing and barrier sheets use where air and moisture permeable properties are essential as well as thermal insulation. By optimizing the component of different fillers, properties of the microporous materials could be adjusted to meet specific requirements.

## Figures and Tables

**Table 1:** Specifications of aerogel particles used in the study.

Properties	Aerogel Powder
Majority particle size range	2~40 μm
Pore diameter	~20 nm
Porosity	90~95%
Surface area	600~800 m^2^/g
Particle density	120~150 kg/m^3^
Thermal conductivity	0.012 W m^−1^ K^−1^ at 25 °C

**Table 2:** Description of the electro-sprayed materials and spinning liquids used for electro-spraying.

Sample Code	Component	Spinning Liquids Used for Electro-Spraying	
		Basic Solution	Fillers
T	PTFE	Teflon^TM^ PTFE 30 aqueous dispersion	None
T/C	PTFE + PCM		PCM (3 g/L)
T/AG	PTFE + aerogel granule		Aerogel granule (3 g/L)
T/AP	PTFE + aerogel powder		Aerogel powder (3 g/L)
T/C/AG	PTFE + PCM +aerogel granule		PCM (3 g/L) + Aerogel granule (3 g/L)
T/C/AP	PTFE + PCM + aerogel powder		PCM (3 g/L) + Aerogel powder (3 g/L)

Note: T—Teflon, C—PCM, AG—Aerogel granule, AP—Aerogel powder.
